# Impact of Tumor Response After Neoadjuvant Treatment on Overall Survival Among Patients With Pancreatic Ductal Adenocarcinoma: National Cancer Database Analysis

**DOI:** 10.7759/cureus.73524

**Published:** 2024-11-12

**Authors:** Megan L Sulciner, Mandisa Bailey, Mengyuan Ruan, Mark Fairweather, Thomas E Clancy, Stanley W Ashley, Jason S Gold, Jiping Wang, George Molina

**Affiliations:** 1 Department of Surgery, Brigham and Women's Hospital, Harvard Medical School, Boston, USA; 2 Center for Surgery and Public Health, Brigham and Women's Hospital, Harvard Medical School, Boston, USA; 3 Department of Surgical Oncology, Dana-Farber / Brigham and Women's Cancer Center, Harvard Medical School, Boston, USA; 4 Surgical Oncology, Dana-Farber / Brigham and Women's Cancer Center, Harvard Medical School, Boston, USA; 5 Department of Surgical Service, VA Boston Healthcare System, West Roxbury, USA

**Keywords:** neoadjuvant therapy, pancreatic cancer, pathologic complete response, surgical resection, tumor downstaging

## Abstract

Background

Complete pathologic response following neoadjuvant therapy (NAT) for pancreatic ductal adenocarcinoma (PDAC) is rare; alternative markers associated with survival are needed. The aim of this study was to evaluate the impact of tumor response to NAT on overall survival (OS) in PDAC patients who received NAT and curative-intent surgery.

Methods

A retrospective study utilizing the 2006-2018 National Cancer Database identified 6,960 adult patients with PDAC who received NAT. As a comparator group, 15,799 patients who underwent upfront surgical resection were separately analyzed. Primary outcome among patients who received NAT was OS according to changes in pathologic T and N staging compared to clinical T and N staging following NAT, defined as favorable response (downstaging) and non-favorable response (no change and upstaging).

Results

After NAT, 35.1%, 43.4%, and 21.5% of patients had T downstaging, no change, and upstaging, respectively. Comparatively, 3.5%, 53.4%, and 43.1% of patients who underwent upfront surgical resection were over-staged, accurately staged, and under-staged, respectively, in reference to the T stage. Adjusting for patient, hospital, treatment, tumor, and margin status covarities, a favorable response to NAT, or T downstaging, was significantly associated with higher OS (HR 0.80, 95% CI 0.75-0.86; median OS 34.4 months, 95% CI 32.6-36.5) compared with a non-favorable response to NAT as the reference group (median OS 27.9 months, 95% CI 26.9-28.8). Similarly, a favorable response to NAT in the N stage was associated with a higher OS (HR 0.87, 95% CI 0.79-0.95; median OS 33.7 months, 95% CI 31.4-36.5) compared with a non-favorable response (median OS 29.3 months, 95% CI 28.6-30.3).

Conclusion

A favorable response to NAT is associated with higher OS among PDAC patients who underwent curative intent surgery.

## Introduction

Pancreatic cancer is the third most common cause of cancer-related death in the United States [1]. Although most patients present with unresectable or metastatic disease, up to 15% of patients present with localized disease amenable to curative-intent surgical resection [2]. Until recently, standard-of-care for pancreatic cancer patients with resectable disease included upfront resection followed by adjuvant therapy. However, the use of neoadjuvant therapy (NAT) has recently become more widespread due to its potential benefit in improving overall survival (OS) by tumor downstaging [3]. A growing body of research on NAT has revealed that longer duration of NAT [4], and multi-agent chemotherapy or chemoradiation [5] are associated with improved survival outcomes. Moreover, survival is also influenced by the achievement of a pathologic complete response (pCR).

Unfortunately, pCR among patients with pancreatic ductal adenocarcinoma (PDAC) who received NAT is rare. Cloyd et al. demonstrated a pCR rate of 3.1% in a cohort of 7,902 patients, while Sell and colleagues had a pCR rate of 0.8% [6,7]. In a single institution study, pCR among patients with borderline resectable and locally advanced disease who underwent NAT and curative-intent resection was demonstrated in only 7.4% of patients [8]. Patients who did have a pCR had improved survival compared to patients with a pathologic partial response (pPR), as the median overall survival was not reached among the pCR patients compared to 33.9 months among patients with a pPR.8 A recent study found that patients who received NAT had improved survival compared to patients who had upfront surgical resection, suggesting the survival benefit may be secondary to downstaging [3]. Given the rarity of pCR, alternative factors that are associated with survival are needed.

The aim of this study was to evaluate the impact of tumor downstaging on overall survival after NAT and curative-intent surgical resection for PDAC using the National Cancer Database (NCDB). Our hypothesis was that among patients who were treated with NAT and underwent surgical resection, pathologic tumor downstaging, indicating a favorable response to NAT, was associated with a higher OS. One significant challenge in using retrospective data to answer this question is the possibility of inaccurate clinical under-staging that is subsequently interpreted as tumor downstaging in the pathologic setting after surgical resection. As such, a secondary aim of this study was to determine the incidence of inaccurate clinical staging among patients who underwent upfront curative intent surgery and to compare this with NAT treatment changes among patients who received NAT followed by curative intent surgery.

## Materials and methods

Study design and patient population

This was a retrospective cohort study that used deidentified patient data obtained from the NCDB. The NCDB is a joint project of the Commission on Cancer of the American College of Surgeons and the American Cancer Society. This is a widely used database that captures more than 70% of all new cancer diagnoses in the United States. The data used in the study are derived from a de-identified NCDB file. The American College of Surgeons and the Commission on Cancer have not verified and are not responsible for the analytic or statistical methodology employed, or the conclusions drawn from these data by the investigator. 

**Figure 1:**
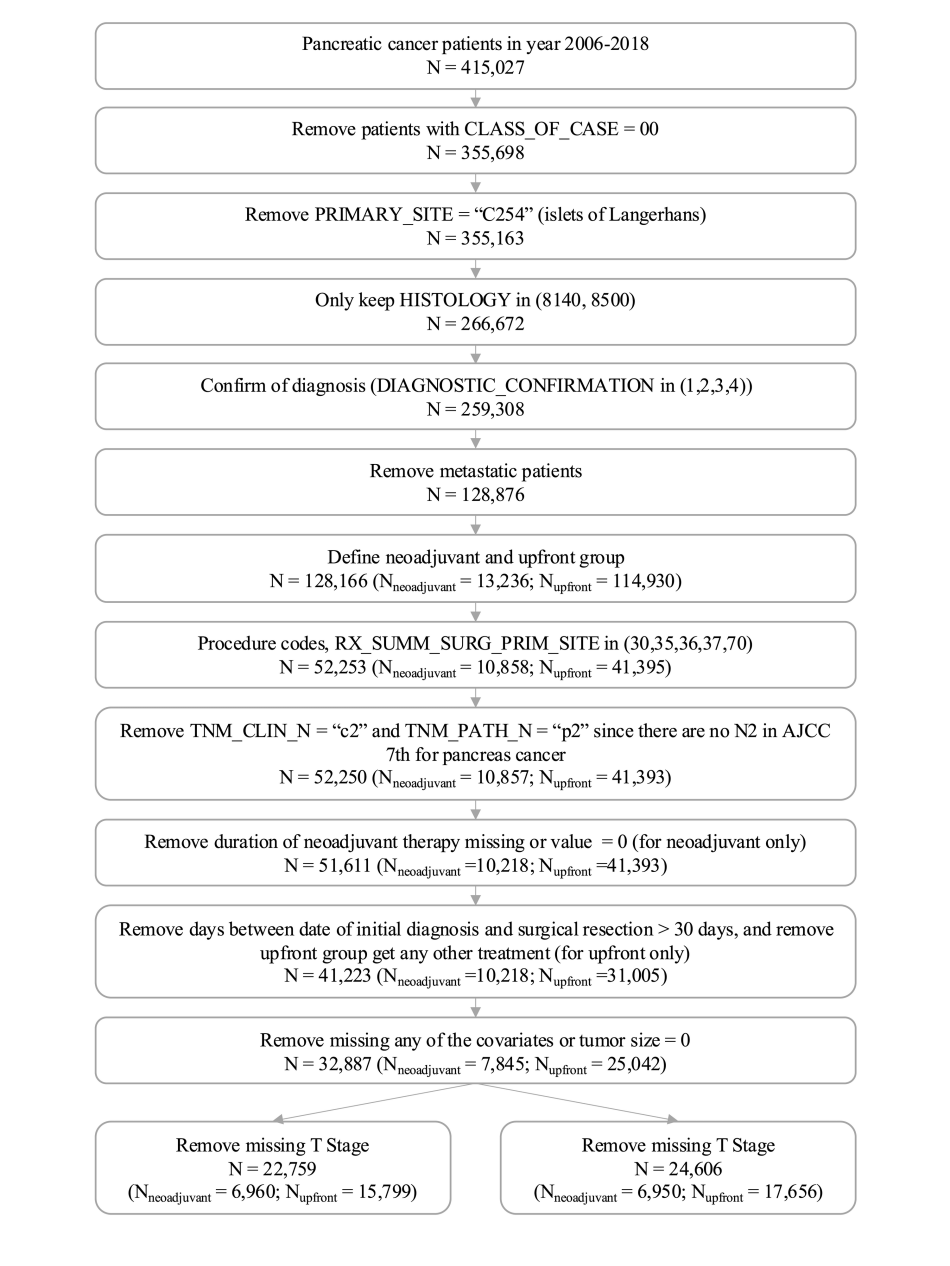
Data cleaning CONSORT diagram CONSORT: Consolidated Standards of Reporting Trials National Cancer Database (NCDB) Data Dictionary Reference - HISTOLOGY: 8140: Adenocarcinoma, NOS; 8500: Invasive carcinoma of no special type DIAGNOSTIC_CONFIRMATION: 1: Positive Histology; 2: Positive Cytology; 3: Positive Histology PLUS positive immunophenotyping and/or positive genetic studies; 4: Positive microscopic confirmation; method not specified; RX_SUMM_SURG_PRIM_SITE: 30: Partial pancreatectomy, NOS; example: distal; 35: Local or partial pancreatectomy and duodenectomy; 36: Local or partial pancreatectomy and duodenectomy WITHOUT distal/partial gastrectomy; 37: Local or partial pancreatectomy and duodenectomy WITH partial gastrectomy (Whipple); 70:  Extended pancreatoduodenectomy

Patients who had a confirmed diagnosis of PDAC during 2006-2018 were identified for this study. Patients with primary tumor sites indicated as “islets of Langerhans”, metastatic disease, stage IV disease, “unknown” disease status, radiation as the only treatment administered during neoadjuvant or adjuvant therapy, and patients with unknown T or N stage were excluded (Figure 1). Patient, treatment, and facility characteristics included patient age at the time of diagnosis, clinical stage at diagnosis, pathologic stage after surgical resection, sex, race, insurance type, Charlson-Deyo score, median income, receipt of adjuvant therapy, tumor size, tumor differentiation, margin status, and facility type. Tumor size demonstrated skewed distribution, therefore this variable was log-transformed. Patients with missing data for the above covariates of interest or a tumor size of “0” were excluded. The clinical and pathologic T stage for PDAC was defined by the American Joint Committee on Cancer (AJCC) Cancer Staging Manual 7th (years 2006-2017) and 8th editions (year 2018).

Patients were then separated into two treatment groups, (1) patients with upfront curative intent surgery alone or (2) patients who received NAT followed by curative intent surgery. Among group 1, patients with greater than 30 days between initial diagnosis and upfront curative-intent resection were excluded to exclude patients who might have developed more advanced disease due to a delay in undergoing surgery [9]. Among group 2, patients who had missing data for the duration of NAT were excluded (Figure 1).

Patients who received upfront curative intent surgery

Patients were then further categorized based on the trajectory of the clinical T or N stage to the pathologic T or N stage. Patients with a higher pathologic stage compared to the clinical stage after curative intent surgery were identified as having been “under-staged”. Patients with a lower pathologic stage compared to the clinical stage after curative intent surgery were identified as having been “over-staged”. Patients with the same pathologic and clinical stages were considered to be “accurately staged”.

Patients who received NAT followed by curative intent surgery

Patients were then further categorized based on the trajectory of the clinical T or N stage to the pathologic T or N stage. Patients with a higher pathologic stage compared to the clinical stage after curative-intent resection were identified as having had “upstaging”. Patients with the same pathologic and clinical stages were considered “no change”.

The primary outcome was to evaluate the association between tumor response and OS among patients with PDAC who received NAT followed by curative intent surgery. In order to determine the relationship between tumor response to NAT and its association with OS, we compared OS between patients who received NAT followed by surgical resection and were identified as having had a favorable response to patients who had a non-favorable response after NAT and surgical resection. 

Statistical analysis

Patient age was reported as a median (IQR). Additional patient characteristics included sex, race, Hispanic ethnicity, insurance type, Charlson-Deyo score, median income, facility type, adjuvant therapy, tumor differentiation, tumor size (IQR), and negative margin status. Comparison of patient characteristics between treatment groups was evaluated by the Wilcoxon t-test for continuous variables and the Chi-square test for categorical variables. Multivariable Cox-proportional hazard models were used to determine survival benefits among T and N stage groups with non-favorable responses, which included no change and upstaging, as the reference for comparison. Survival was reported as hazard ratio (HR) with 95% confidence intervals. Kaplan-Meier survival curves were used to compare OS between treatment groups and response groups. OS was defined as the date of initial diagnosis to the date of last follow-up or death. Sensitivity and specificity of N stage response with a favorable response in T stage as the standard was performed. All analyses were performed using R statistical software (v4.1.0: R Core Team 2021, R Foundation for Statistical Computing, Vienna, Austria).

This retrospective cohort study was approved by the Institutional Review Board of Brigham and Women’s Hospital in Boston, MA (Protocol #: 2022P000974).

## Results

A total of 22,759 adult patients (minimum 18 years of age) with histology-confirmed pancreatic ductal adenocarcinoma (PDAC) who underwent curative intent resection between 2006 and 2018 in the United States were included in this study. Among these patients, 6,960 patients received NAT followed by curative intent surgical resection and 15,799 patients received curative intent surgical resection as their initial treatment. The median age for the NAT group was 65 (Table 1). 48.5% (N=11150) of the patients in the NAT group were female. The overall distribution of NAT patients identified as White was 88.1% (N=6134), Black 8.7% (N=604), and Hispanic 4.1% (N=283). Most NAT patients had private insurance (43.2%, N=3005) or Medicare (49.2%, N=3424). The majority of NAT patients had a Charlson-Deyo score of 0 (64.9%, N=4517). 14.7% (N=1020) of patients had a median income of less than $40,227, whereas 39.4% (N=2740) had a median income of greater than or equal to $63,333. Most NAT patients received care at an academic/research program (63.9%, N=4445).

**Table 1:** Characteristics of patients with pancreatic ductal adenocarcinoma, overall and by treatment group. The data for patient sex, race, insurance type, Charlson-Deyo score, median income, facility type, receipt of adjuvant therapy, tumor differentiation, and margin status are represented as N (%). Patient age and tumor size are represented as median with interquartile range. P-value < 0.05 is significant.

		Treatment Group		
	Overall (N=22759)	Neoadjuvant (N=6960)	Upfront (N=15799)	p-value
Age, median (IQR)	67.0 (59.0-73.0)	65.0 (58.0-71.0)	67.0 (60.0-74.0)	<0.001
Female, N (%)	11150 (49.0%)	3377 (48.5%)	7773 (49.2%)	0.35
Race, N (%)				0.02
White	19866 (87.3%)	6134 (88.1%)	13732 (86.9%)	
Black	2077 (9.13%)	604 (8.7%)	1473 (9.3%)	
Other	816 (3.6%)	222 (3.2%)	594 (3.8%)	
Hispanic, N (%)	1036 (4.6%)	283 (4.1%)	753 (4.8%)	0.02
Insurance type, N (%)				<0.001
Not insured	408 (1.8%)	87 (1.3%)	321 (2.0%)	
Private Insurance / Managed Care	8713 (38.3%)	3005 (43.2%)	5708 (36.1%)	
Medicaid	1088 (4.8%)	333 (4.8%)	755 (4.8%)	
Medicare	12269 (53.9%)	3424 (49.2%)	8845 (56.0%)	
Other Government	281 (1.2%)	111 (1.6%)	170 (1.1%)	
Charlson-Deyo Comorbidity score, N (%)				0.63
0	14675 (64.5%)	4517 (64.9%)	10158 (64.3%)	
1	5965 (26.2%)	1800 (25.9%)	4165 (26.4%)	
2	1425 (6.3%)	442 (6.4%)	983 (6.2%)	
≥3	694 (3.1%)	201 (2.9%)	493 (3.1%)	
Median income, N (%)				0.002
< $40,227	3549 (15.6%)	1020 (14.7%)	2529 (16.0%)	
$40,227 - $50,353	4773 (21.0%)	1534 (22.0%)	3239 (20.5%)	
$50,354 - $63,332	5296 (23.3%)	1666 (23.9%)	3630 (23.0%)	
≥$63,333	9141 (40.2%)	2740 (39.4%)	6401 (40.5%)	
Facility type, N (%)				<0.001
Community Cancer Program	511 (2.3%)	126 (1.8%)	385 (2.4%)	
Comprehensive Community Cancer Program	5529 (24.3%)	1234 (17.7%)	4295 (27.2%)	
Academic/Research Program	12744 (56.0%)	4445 (63.9%)	8299 (52.5%)	
Integrated Network Cancer Program	3975 (17.5%)	1155 (16.6%)	2820 (17.8%)	
Adjuvant, N (%)	13743 (60.4%)	2638 (37.9%)	11105 (70.3%)	<0.001
Tumor differentiation, N (%)				<0.001
Well	1838 (8.1%)	530 (7.6%)	1308 (8.3%)	
Moderately	10789 (47.4%)	2567 (36.9%)	8222 (52.0%)	
Poor/Undifferentiated	7099 (31.2%)	1429 (20.5%)	5670 (35.9%)	
Unknown	3033 (13.3%)	2434 (35.0%)	599 (3.79%)	
Tumor Size (mm), median (IQR)	30.0 (25.0-40.0)	31.0 (25.0-40.0)	30.0 (25.0-40.0)	0.18
Negative tumor margins, N (%)	18071 (79.4%)	5795 (83.3%)	12276 (77.7%)	<0.001

PDAC patients who received upfront curative intent resection (upfront group) had a median age of 67 (Table 1). 49.2% (N=7773) were female, equivalent to the distribution of female patients in the NAT group. 86.9% (N=13732) of patients identified as White, 9.3% (N=1473) identified as Black, and 4.8% (N=753) identified as Hispanic. Over half of the patients in the upfront group had Medicare insurance (56.0%, N=8845). The distribution of the Charlson-Deyo score between the NAT group and the upfront group was not significantly different. While comparisons of age, race, Hispanic ethnicity, insurance type, and median income were statistically significant between NAT and upfront groups, the differences were not clinically meaningful. However, compared to the NAT group, fewer patients who underwent upfront surgical resection received their care at an academic/research program (52.5% versus 63.7%) (p<0.001). Unsurprisingly, 70.1% (N=11105) of patients in the upfront group received adjuvant therapy, significantly more than the NAT group (37.9%, 2638) (p<0.001). Patients in the upfront group were more likely to have moderately differentiated tumors (52.0%, N=8222) compared to the NAT group (36.9%, N=2567) (p<0.001). Notably, more patients in the upfront group (35.9%) had poor/undifferentiated tumors compared to patients in the NAT group (20.5%, N=1429). There was no statistical difference in tumor size between the groups. However, patients in the NAT group were more likely to achieve negative margins (83.8% versus 77.7% in the upfront group, p<0.001).

Tumor response to neoadjuvant therapy compared to upfront resection

**Figure 2:**
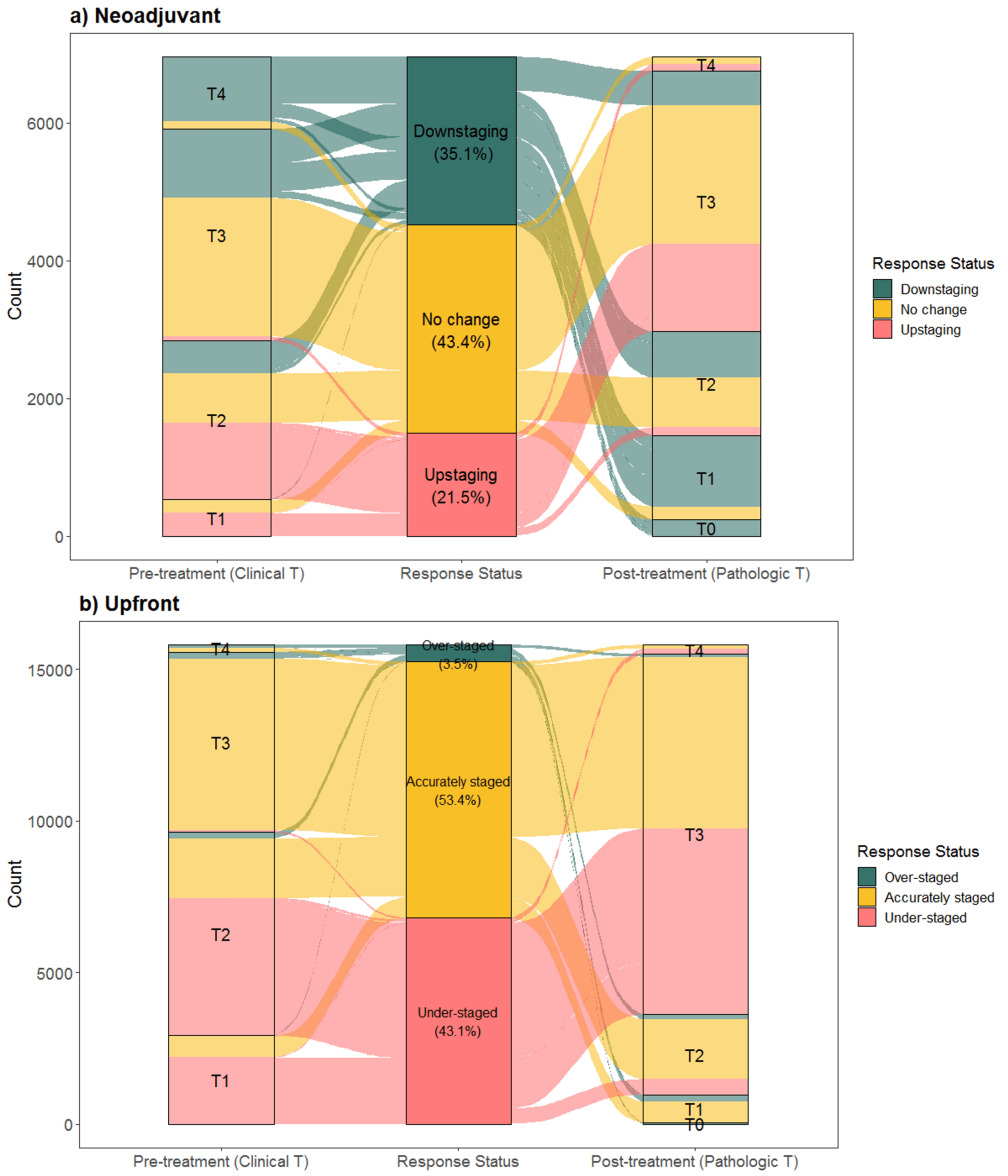
Sankey plot representation of clinical to pathologic change for pancreatic ductal adenocarcinoma cancer patients. Patients who received a) neoadjuvant treatment demonstrated downstaging (35.1%), no change (43.4%), and upstaging (21.5%) compared to b) upfront treatment with patients who were over-staged (3.5%), accurately staged (53.4%), and under-staged (43.1%).

To study the association between tumor response and OS after NAT and curative intent surgical resection for PDAC, we first sought to determine the trend between the clinical stage prior to resection and the pathologic stage after resection using a Sankey plot (Figure 2). Based on the trend of clinical to pathologic T stage, tumor response was categorized as downstaging, no change, or upstaging. For patients in the NAT group, 35.1% had clinical to pathologic downstaging, 43.4% had no change, and 21.5% had upstaging, respectively (Figure 2a). Conversely, in the upfront group, 3.5% of patients were over-staged, 53.4% were accurately staged, and 43.1% were under-staged (Figure 2b). For clinical to pathologic N stage, 11.8% in the NAT group demonstrated downstaging compared to 1.2% of patients in the upfront group who were over-staged. Pathologic staging for all patients was defined by both the AJCC 7th and 8th editions given the inclusion timeframe of this study. Given the differences in T and N staging definitions between AJCC editions, even when patients defined by AJCC 8th edition were excluded, 32.5% of patients in the NAT group had downstaging, and 3.1% in the upfront group were over-staged. Similarly, 12.2% of patients in the NAT group had N downstaging, compared to 1.2% in the upfront group who were over-staged.

Since the focus of our study was tumor response to NAT, patients with “no change” or “upstaging” were considered as having a “non-favorable” response to NAT. Patients with a lower pathologic stage compared to the clinical stage (i.e., “downstaging”) after curative-intent resection were identified as having had a “favorable” response to NAT.

Survival outcomes based on tumor response to NAT

**Figure 3:**
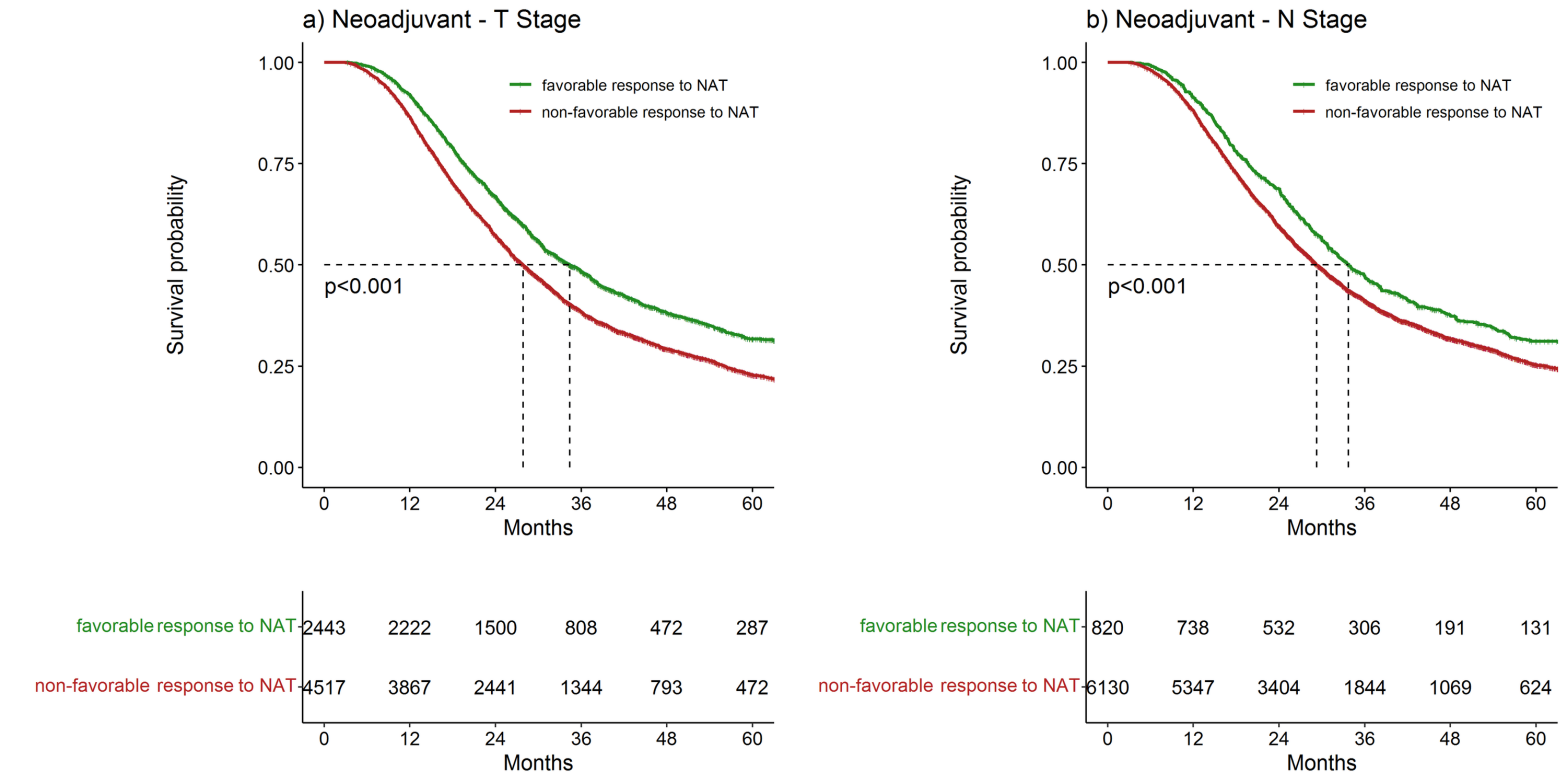
Overall survival for patients who received neoadjuvant therapy (NAT) based on change in clinical to pathologic stage. Response to neoadjuvant in a) T-Stage demonstrated a median overall survival of 34.4 months for patients with a favorable response (green line) compared to 27.9 months with a non-favorable response (red line) (p<0.001). Response to neoadjuvant in b) N-Stage demonstrated a median overall survival of 33.7 months for patients with a favorable response (green line) compared to 29.3 months with a non-favorable response (red line) (p<0.001). P-value < 0.05 is significant.

We then sought to evaluate OS based on response to NAT. Among patients that received NAT, median post-diagnosis OS was significantly higher in patients with a favorable response in the T stage compared to those with a non-favorable response (Figure 3a). Similarly, patients with a favorable response in the N stage also had a higher median OS compared to those with a non-favorable response in the N stage (Figure 3b).

After adjusting for age, sex, race, Charlson-Deyo score, median income, facility type, receipt of adjuvant therapy, duration of neoadjuvant therapy, tumor size (log-transformed), tumor differentiation, and margin status, favorable response in T stage among patients that received NAT followed by surgical resection was associated with a higher OS as compared to a non-favorable response (HR 0.80, 0.75-0.86) (p<0.001) (Table 2). Similarly, a favorable response in N staging was associated with higher OS compared to a non-favorable response (HR 0.87, 0.79-0.95) (p=0.003) (Table 2). To exclude the possibility of staging differences between patients defined by the AJCC 7th edition or 8th, the exclusion of patients from 2018 was performed for a sensitivity analysis. After excluding patients from 2018, the post-diagnosis survival was not significantly different from the initial findings - a favorable response to NAT in T stage (HR 0.84, 0.78-0.90) and N stage (HR 0.88, 0.80-0.97) continued to be associated with significantly higher OS (p<0.001 and p=0.009 respectively) (Table 3).

**Table 2:** Multivariable post-diagnosis survival among patients with pancreatic adenocarcinoma who received neoadjuvant therapy. NAT: Neoadjuvant therapy Models were adjusted for age, sex, race, Charlson-Deyo score, median income, facility type, adjuvant therapy, duration of neoadjuvant therapy, tumor size (log-transformed), tumor differentiation, and margin status. The data is represented as HR (95% CI), p-value <0.05 is significant.

Response Status	T Stage			N Stage	
	HR (95% CI)	p-value		HR (95% CI)	p-value
Non-favorable response to NAT	1 (ref.)	--		1 (ref.)	--
Favorable response to NAT	0.80 (0.75, 0.86)	<0.001		0.87 (0.79, 0.95)	0.003

**Table 3:** Sensitivity analysis, multivariable post-diagnosis survival among patients with pancreatic adenocarcinoma who received neoadjuvant therapy during 2006-2017. Models were adjusted for age, sex, race, Charlson-Deyo score, median income, facility type, adjuvant therapy, duration of neoadjuvant therapy, tumor size (log-transformed), tumor differentiation, and margin status. The data is represented as HR (95% CI), p-value < 0.05 is significant.

Response Status	T Stage			N Stage	
	HR (95% CI)	p-value		HR (95% CI)	p-value
Non-favorable response to NAT	1 (ref.)	--		1 (ref.)	--
Favorable response to NAT	0.84 (0.78, 0.90)	<0.001		0.88 (0.80, 0.97)	0.009

In order to address selection bias in our NAT group, we aimed to delineate the proportion of all patients who received NAT and underwent curative-intent resection. Downstaging was most prominent in our NAT cohort in patients with clinical T4 stage, thus we determined the percentage of all clinical T4 stage patients who received NAT and then had surgery. A total of 1,676 patients with clinical T4 stage PDAC who received NAT were identified, with 88.7% of these patients receiving curative intent surgery. The distribution of patients with clinical T4 disease that received NAT and then underwent curative-intent surgery by pathologic T stage was relatively similar for pathologic stage T0 (87.3%, N=89/102), T1 (86.6%, N=265/306), T2 (81.9%, N=230/281), and T3 (84.1%, N=671/798). Comparatively, 76.7% (N=145/189) of patients with no change in clinical to pathologic T4 disease after NAT underwent curative-intent resection (Table 4).

**Table 4:** Percentage of patients with clinical T4 PDAC who received curative-intent surgery after NAT by pathology response in T stage. ^a^Patients who received NAT after the data cleaning step 7 in Figure 1. ^b^Curative-intent surgery included RX_SUMM_SURG_PRIM_SITE in (30, 35, 36, 37, 70) NAT: neoadjuvant therapy; PDAC: pancreatic ductal adenocarcinoma

Group	Pathological T	N^a^	% of curative-intent surgery^b^
NAT Clinical T4 (N = 1676)	T0	102	89 (87.3%)
	T1	306	265 (86.6%)
	T2	281	230 (81.9%)
	T3	798	671 (84.1%)
	T4	189	145 (76.7%)

We next sought to evaluate for an association between response to NAT in T with response in the N stage. A chi-square analysis demonstrated that a response to NAT in the T stage was significantly associated with a response in the N stage (p<0.001). To further elucidate the relationship between a response in the T stage with a response in the N stage, a favorable response in the T stage was used as the reference standard, and we performed a sensitivity and specificity analysis. A favorable response in the T stage was not associated with a favorable response in the N stage. However, a non-favorable response in the T stage was associated with a non-favorable response in the N stage. The sensitivity of response in the T stage was 16% and the specificity was 97% in predicting the N stage response.

## Discussion

The findings in our study demonstrate that tumor downstaging among patients who received NAT and underwent surgical resection may be a surrogate marker for tumor response to NAT. Using the NCDB, we found that a favorable response to NAT, as defined by downstaging, among patients who received NAT and underwent curative intent surgery was associated with higher OS compared to patients with a non-favorable response. Moreover, these findings were applicable to both T and N staging, such that favorable response in either the T stage or N stage was associated with higher OS.

The ideal tumor response to NAT is pCR. Although pCR after NAT and curative intent surgery has been associated with higher OS, [7, 10] pCR is rare. Studies have estimated that pCR in PDAC occurs in up to only 3.9% of patients who received NAT [6,11]. Prior to the implementation of NAT, few patients presented with tumors amenable to resection. Several studies have now demonstrated that the use of NAT in the treatment of borderline resectable or locally advanced PDAC is associated with a higher likelihood of achieving an R0 resection [12,13]. However, given the limited number of patients who ultimately achieve pCR, the identification of additional surrogate markers for tumor response is needed.

Several studies have focused on quantifying tumor response to NAT with a distinction on major pathologic response. Major pathologic response based on histologic characteristics (percent fibrosis compared to percent of viable tumor cells) has been associated with improved survival among patients with PDAC who received NAT [14]. Recent results of the phase II SWOG S1505 clinical trial demonstrated that a major pathologic response for low-grade tumors (grades 1-2) was achieved in 33% of patients regardless of the type of NAT regimen that was used [15]. In the cohort of this current study that included patients with all tumor grades and T stages, we found that 35.1% of patients who received NAT had downstaging on pathologic assessment. Furthermore, among patients with clinical T4 stage disease who received NAT over 80% underwent curative intent resection. Moreover, there is data to suggest that NAT for upfront resectable disease may also improve local disease control and as such, decrease rates of recurrence [16-18]. Taken together, these findings support the use of NAT for all T stages.

While our results demonstrate that a favorable response to NAT is associated with higher OS, we also found that a favorable response in the N stage was also associated with higher OS. Previous studies have shown that NAT was associated with higher rates of negative pathologic nodal disease [3,19,20]. Moreover, negative nodal status was associated with improved overall survival, particularly in patients with locally advanced or borderline resectable disease [21,22]. Our data demonstrates that a favorable response in the N stage, inclusive of all clinical N stages, was associated with higher OS compared to patients with a non-favorable response. Therefore, N downstaging alone may be indicative of tumor response to NAT.

Clinical nodal status often underestimates pathologic disease extent. In fact, patients who received upfront resection with clinical N0 were more often upstaged [23]. Particularly, in patients with higher pathologic T stage (stage 3) with a nodal status of N1, disease extent was more likely under-estimated [23]. Studies have demonstrated that even in early-stage disease (I-II), NAT should be considered since up to 38% of patients demonstrate nodal downstaging [24]. Furthermore, nodal downstaging after NAT in patients with T stage I-III was associated with improved OS [25]. While our data demonstrated that a favorable response in the T stage was not indicative of a congruent response in the N stage, a non-positive response in T was significantly associated with a non-favorable nodal response. (Data not shown)

Limitations

This study has certain limitations. First, this is a retrospective study, and it is not possible to definitively identify inaccurate clinical staging. To address this limitation, we compared patients who received NAT followed by surgery to patients who underwent upfront surgical resection. We found that the proportion of patients that underwent upfront surgical resection and had over-staging was significantly lower than the proportion of patients in the NAT group that had downstaging. This finding provides confidence that downstaging among patients who received NAT followed by surgery may be a reliable marker of tumor response. Second, given our NAT group only included patients who received curative intent resection, we sought to address immortal time bias by determining the percentage of patients who underwent surgery among all patients who received NAT. We found that the majority of patients with clinical T4 disease who received NAT did undergo surgical resection, thus demonstrating that selection bias is most likely not influencing our findings. (Table 5) Third, NCDB does not capture patients who received their cancer care at hospitals that are not accredited by the Commission on Cancer, thus potentially limiting the generalizability of the findings. However, NCDB captures 70% of all new cancer diagnoses in the United States and it is a commonly used database. Additionally, the main outcome of interest was OS, and this encompasses all-cause mortality and is therefore not exclusive to PDAC disease-specific survival. Finally, the duration of the study included nodal staging defined by both the AJCC 7th and 8th editions, however, N2 data was only available in the AJCC 8th edition, thus limiting the number of patients that could have been categorized with N2 disease. However, to account for discrepancies between the AJCC editions, we adjusted for tumor size within our multi-variable analysis and performed sensitivity analyses in which we excluded patients defined by the AJCC 8th edition (i.e. patients diagnosed after 2017). These sensitivity analyses revealed no significant differences in our findings.

## Conclusions

Among patients with PDAC who received NAT and underwent curative intent surgery, tumor response was associated with OS. A favorable tumor response, as defined by T and N downstaging, was associated with a higher OS compared to a non-favorable tumor response. This study provides evidence that T or N stage downstaging following NAT and curative intent surgery could be a surrogate for tumor response and OS.
